# Human Neural Stem Cells Over-Expressing VEGF Provide Neuroprotection, Angiogenesis and Functional Recovery in Mouse Stroke Model

**DOI:** 10.1371/journal.pone.0000156

**Published:** 2007-01-17

**Authors:** Hong J. Lee, Kwang S. Kim, In H. Park, Seung U. Kim

**Affiliations:** 1 Brain Disease Research Center, Ajou University School of Medicine, Suwon, Korea; 2 College of Bioscience and Biotechnology, Korea University, Seoul, Korea; 3 Division of Neurology, Department of Medicine, University of British Columbia Hospital, University of British Columbia, Vancouver, Canada; Netherlands Cancer Institute, Netherlands

## Abstract

**Background:**

Intracerebral hemorrhage (ICH) is a lethal stroke type. As mortality approaches 50%, and current medical therapy against ICH shows only limited effectiveness, an alternative approach is required, such as stem cell-based cell therapy. Previously we have shown that intravenously transplanted human neural stem cells (NSCs) selectively migrate to the brain and induce behavioral recovery in rat ICH model, and that combined administration of NSCs and vascular endothelial growth factor (VEGF) results in improved structural and functional outcome from cerebral ischemia.

**Methods and Findings:**

We postulated that human NSCs overexpressing VEGF transplanted into cerebral cortex overlying ICH lesion could provide improved survival of grafted NSCs, increased angiogenesis and behavioral recovery in mouse ICH model. ICH was induced in adult mice by unilateral injection of bacterial collagenase into striatum. HB1.F3.VEGF human NSC line produced an amount of VEGF four times higher than parental F3 cell line in vitro, and induced behavioral improvement and 2–3 fold increase in cell survival at two weeks and eight weeks post-transplantation.

**Conclusions:**

Brain transplantation of F3 human NSCs over-expressing VEGF near ICH lesion sites provided differentiation and survival of grafted human NSCs and renewed angiogenesis of host brain and functional recovery of ICH animals. These results suggest a possible application of the human neural stem cell line, which is genetically modified to over-express VEGF, as a therapeutic agent for ICH-stroke.

## Introduction

Intracerebral hemorrhage (ICH) represents at least 15% of all strokes in the western population and a considerably higher proportion at 50–60% in the oriental populations in Korea, Chin and Japan [1], [2]. ICH is a lethal stroke type, as mortality approaches 50% and neurological disability in survivors is common. Since medical therapy against ICH such as mechanical removal of hematoma, prevention of edema formation by drugs, and reduction of intracranial pressure, shows only limited effectiveness, alternative approach is required such as stem cell-based cell therapy [3], [4].

Recent progresses in stem cell biology have opened up an avenue to therapeutic strategies to replace lost neural cells by transplantation of neural stem cells (NSCs) in CNS injury and disease [5]–[7]. Previous studies have indicated that NSCs or neural progenitor cells engrafted in animal models of stroke survive and ameliorate neurological deficits in the animals [8]–[13]. Among these studies, human neural progenitor cells isolated from fetal brain have been transplanted into the brain of stroke animal models and found to restore brain function [8], [13]. This approach, however, is not widely acceptable for stroke patients because of moral, religious and logistic problems associated with the use of human fetal tissues. In addition, primary human NSCs derived from fetal tissues can be provided for only a limited time before they undergo senescence, and it is difficult to secure sufficient numbers and homogeneous populations of human NSCs from fetal brain. These problems can be circumvented by the use of stable, permanent cell lines of human NSCs.

Previously we have generated a clonal permanent human neural stem cell line, HB1.F3 (F3), that had been immortalized via a retroviral vector encoding v-myc oncogene [14], [15], and this F3 human NSC line shows multipotent capacity to differentiate into neurons and glial cells [15]–[17], ameliorate neurological deficits in animal models of Parkinson disease [18], Huntington disease [19], [20] and lysosomal storage disease [21] following their transplantation into the brain. In stroke animal models, intravenously transplanted F3 human NSCs migrated selectively to the damaged brain sites caused by ischemia [9]–[11] and ICH [12], differentiated into neurons and astrocytes, and promoted functional recovery in these animals. However, low survival rate of grafted F3 NSCs in ischemia and ICH rats in the previous studies is a grave concern; less than 50% of grafted NSCs survived in ICH mice at 2 weeks post-transplantation.

One significant way to promote differentiation and survival of transplanted NSCs is to modulate microenvironment in the injured brain following ICH, and this might be accomplished by supplying additional neurotrophic growth factors such as brain-derived neurotrophic factor (BDNF), glial cell line-derived neurotrophic factor (GDNF) or vascular endothelial growth factor (VEGF) which are known to play key roles in proliferation, differentiation and survival of NSCs. VEGF is one of such growth factors which could be used in combination with transplanted NSCs to improve therapeutic efficiency of cellular transplantation. VEGF is an angiogenetic growth factor acting as a potent mitogen and survival factor specific to endothelial cells [22], [23], and also known for neuroprotective effect against ischemic injury [24]–[28].

Considering evidence of functional recovery in stroke animal models following brain transplantation of human NSCs [9]–[12] and VEGF treatment [24]–[28], we wished to investigate whether the human NSCs overexpressing VEGF, by pairing clonal human NSCs with VEGF gene can lead to the increased cell survival and functional improvement in mouse ICH stroke model.

## Materials and Methods

### Clonal human neural stem cell line

Telencephalon tissue from a 15 week gestational human fetal brain was utilized to generate primary cell culture from which immortalized cell lines of human neural stem cells (NSCs) were generated using a retroviral vector encoding v-myc oncogene [14], [15]. One of the NSC clones, HB1.F3, expressed phenotypes specific for neural stem cells including ABCG2 and nestin. Additionally, F3 cells differentiated into neurons and expressed Na^+^ current when cells were transduced with NeuroD gene [16]. F3 NSCs were grown in a serum-free medium (DM4) consisting of Dulbecco's modified Eagle medium with high glucose (DMEM) containing 10 µg/ml insulin, 10 µg/ml transferrin, 30 nM sodium selenate, 50 nM hydrocortisone, 0.3 nM triiodothyronine and 20 µg/ml gentamicin [29]. Recombinant human bFGF (10 ng/ml; PeproTech, Rocky Hill, NJ) was supplemented to the DM4 during the routine feeding. All chemicals except bFGF were obtained from Sigma (St Louis, MO).

### Transfection of VEGF gene into F3 NSCs

**Figure 1:**
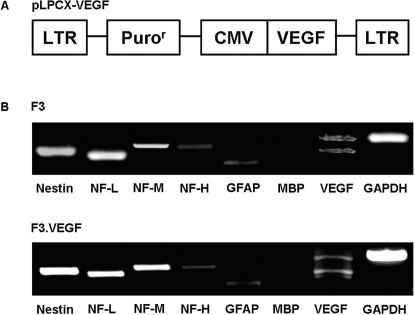
A: The retroviral vector encoding VEGF (pLPCX.VEGF) used in the present study in the generation of HB1.F3, VEGF human neural stem cell line. B: Gene expression of cell type-specific markers as studied by RT-PCR in F3 and F3.VEGF human neural stem cell lines. Both F3 and F3.VEGF human neural stem cell lines express cell type specific markers NF-L, NF-M and NF-H, an astrocyte marker GFAP and VEGF, while they do not express MBP, a cell type marker of oligodendrocytes.

Plasmid pLPCX.VEGF containing the full length human VEGF cDNA was used in the present study. PG13 mouse packaging cell line was transfected with pLPCX.VEGF vector (Fig. 1A) using LipofectAMINE (Invitrogen, Carlsbad, CA) and a stable PG13 cell line was selected using 10 µg/ml puromycin for 3 days. Replication incompetent retroviral vector collected from PG13.VEGF cells was used for transfection of F3 NSCs. Puromycin-resistant F3.VEGF clones were isolated, screened, and one of the clones F3.VEGF.D2 was expanded, and used for the transplantation. Expression of VEGF in F3.VEGF.D2 cell line was analyzed by RT-PCR, ELISA and laser-scanning confocal microscope.

### Immunocytochemistry

Immunocytochemical demonstration of VEGF protein in F3.VEGF human NSCs was performed as described previously [30]. F3.VEGF human NSCs plated on poly-L-lysine-coated Aclar plastic coverslips (9 mm in diameter, SPL, Seoul, Korea) were grown in DM4 serum-free medium supplemented with bFGF for 2–5 days, rinsed in PBS and fixed in cold acid alcohol (5% glacial acetic acid in 95% ethanol) for 10 min at −20°C. Cultures were incubated in blocking solution consisted of PBS containing 10% normal goat serum and 3% Triton-X at room temperature (RT) for 20 min, followed by primary antibody specific for human VEGF (1∶200, mouse monoclonal, R&D, Minneapolis, MN) for 24 hr at 4°C, then with Alexa Fluor 594-conjugated anti-mouse IgG (Molecular Probe, Eugene, OR) for 1 hr at RT. Cells were counterstained with 4′, 6-diamino-2-phenylindole (DAPI, Sigma, St Louis, MO) to identify cellular nuclei. Following immunostaining, cells were mounted on glass slides using gelvatol and viewed under an Olympus laser-scanning confocal microscope (Tokyo, Japan).

### RT-PCR analysis

F3 and F3.VEGF human NSCs were collected by centrifugation and total RNA was isolated using Trizol, according to manufacture's protocol (Promega, Madison, WI). One µg of total RNA was reverse-transcribed into first-strand cDNA using oligo-dT primer. Reverse transcription was performed with AMV reverse transcriptase (Takara, Seoul, Korea) for 1 hr at 42°C, inactivated for 10 min at 95°C and cooled to 4°C. The cDNA was diluted to a final volume of 25 µl, and a 2 µl aliquot was used in a PCR reaction containing 1× DNA polymerase buffer, 1.5 mM MgCl2, 0.2 mM dNTPs, 10 pmol primers, 2.5 units of Taq polymerase (Takara). The cDNA was amplified using 30 PCR cycles and RT-PCR products were separated electrophoretically on 1.2% agarose gel containing ethidium bromide and visualized under UV light. The primers used for the RT-PCR for nestin, NF-L, NF-M, NF-H, GFAP, MBP, VEGF, and GAPDH (all human) are listed in Table 1.

**Table 1:**
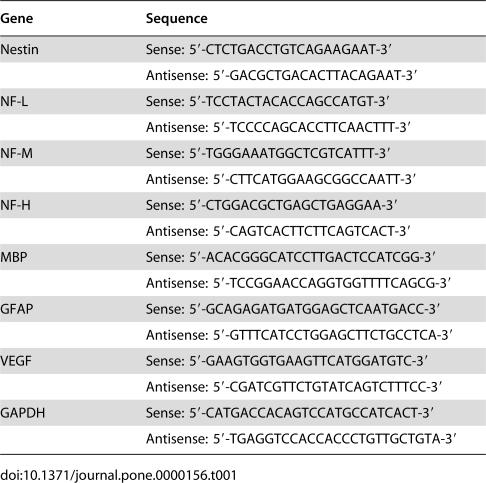
Sequence of PCR primers

Gene	Sequence
Nestin	Sense: 5′-CTCTGACCTGTCAGAAGAAT-3′
	Antisense: 5′-GACGCTGACACTTACAGAAT-3′
NF-L	Sense: 5′-TCCTACTACACCAGCCATGT-3′
	Antisense: 5′-TCCCCAGCACCTTCAACTTT-3′
NF-M	Sense: 5′-TGGGAAATGGCTCGTCATTT-3′
	Antisense: 5′-CTTCATGGAAGCGGCCAATT-3′
NF-H	Sense: 5′-CTGGACGCTGAGCTGAGGAA-3′
	Antisense: 5′-CAGTCACTTCTTCAGTCACT-3′
MBP	Sense: 5′-ACACGGGCATCCTTGACTCCATCGG-3′
	Antisense: 5′-TCCGGAACCAGGTGGTTTTCAGCG-3′
GFAP	Sense: 5′-GCAGAGATGATGGAGCTCAATGACC-3′
	Antisense: 5′-GTTTCATCCTGGAGCTTCTGCCTCA-3′
VEGF	Sense: 5′-GAAGTGGTGAAGTTCATGGATGTC-3′
	Antisense: 5′-CGATCGTTCTGTATCAGTCTTTCC-3′
GAPDH	Sense: 5′-CATGACCACAGTCCATGCCATCACT-3′
	Antisense: 5′-TGAGGTCCACCACCCTGTTGCTGTA-3′

### Mouse ICH model

All experimental procedures were approved by the Animal Care Committee of the Ajou University Hospital. ICH was induced by stereotaxic, intrastriatal administration of bacterial collagenase by previously described methods [12], [31]. In brief, after an intraperitoneal injection of 1% ketamine (30 mg/kg) and xylazine hydrochloride (4 mg/kg), the mice were placed in a stereotaxic frame (Kopf Instruments, Tujunga, CA). A burr hole was made, and a 30-gauge needle was inserted through the burr hole into the striatum (0.1 mm posterior, 4.0 mm ventral, and 2.0 mm lateral to the bregma). ICH was induced by the administration of collagenase type IV (1 µl saline containing 0.078 U, Sigma) over a period of 5 min. After remaining in place for another 3 min, the needle was gently removed. During the procedure, rectum temperature was monitored and maintained at 37±0.5°C

### Brain transplantation

Experimental groups are group 1 (control): injection of vehicle/PBS (2 µl, n = 20); group 2: transplantation of F3 (2×10^5^/2 µl, n = 23); and group 3: transplantation of F3.VEGF (2×10^5^/2 µl, n = 27). At 7 days after ICH, F3 or F3.VEGF NSCs (2×10^5^ cells/2 µl) were transplanted into ipsillateral striatum, 2 mm cranial to the hemorrhagic lesion, calculated from bregma: 0.1 mm anterior and 2.0 mm right lateral to the bregma and 2.0 mm ventral to the cortical surface. In order to detect the grafted cells in host brain, donor F3 and F3.VEGF human NSCs were infected with an adenovirus vector encoding LacZ gene (pAV.LacZ) in vitro at 100 MOI (PU/cell) for 24 hr before transplantation.

### Behavioral Testing

Behavioral testing was performed weekly with the rotarod and modified limb placement tests, which were monitored by 2 individuals blinded to rat treatment status. In the rotarod test [12], the rats were placed on the rotarod cylinder, and the time the animals remained on the rotarod was measured. The speed was slowly increased from 4 to 40 rpm within a period of 5 min. The trial was ended if the animal fell off the rungs or gripped the device and spun around for 2 consecutive revolutions. The animals were trained for 3 days before stem cell transplantation. The maximum duration (in seconds) on the device was recorded with 3 rotarod measurements 1 day before ICH induction. Motor test data are presented as percentages of the maximal duration compared with the internal baseline control (before ICH).

The details of limb placement test have been described previously [32]. Test was repeated 3 times and are scored in the following manner: normal performance, 0 points; performance with a delay (2 seconds) and/or incomplete, 1 point; no performance, 2 points. A total of 9 points means maximal neurological deficit, and 0 points means normal performance. Additionally, the body weights of all animals were checked weekly for 8 weeks.

### Histology and immunohistochemistry

At the end of behavioral testing, each animal was anesthetized and perfused through the heart with cold saline followed by 4% paraformaldehyde in 0.1 M phosphate buffer. The brains were post-fixed in same fixative for 24 hr, followed with cryoprotection in 30% sucrose for 24 hr and then 30 µm sections were prepared on a cryostat. Three sections through the needle entry site, which was identifiable on the brain surface, and sites 1.0 mm anterior and 1.0 mm posterior to plane were processed for β-gal staining to analyze the hemisphere area. These sections are representative of the core of the ICH lesion.

The morphometric analyses involved computer-assisted hand delineation of the area of the striatum, cerebral cortex, and ventricles, as well as the whole hemisphere. Adjacent serial coronal sections were processed for double immunofluorescence staining of β-gal (1∶400, mouse monoclonal, Sigma) and antibodies specific for cell type specific markers. Antibodies specific for neurofilament low molecular weight protein (NF-L, 1∶1000, rabbit, Chemicon), neurofilament high molecular weight protein (NF-H, 1∶1000, rabbit, Chemicon), microtubule associated protein-2 (MAP2, 1∶500, rabbit, Chemicon), glial fibrillary acidic protein (GFAP, 1∶200, rabbit, DAKO, Carpinteria, CA), von Willebrandt factor (vWF, 1∶200, rabbit, Ongogene, Cambridge, MA) and VEGF (1∶200, rabbit, Oncogene) were used for cell type identification of neurons, astrocytes, and endothelial cells. For the identification of grafted F3 and F3.VEGF human NSCs, a monoclonal antibodies specific for human cell nuclear matrix antigen (HuNuA, 1∶200, Chemicon) and human mitochondria (HuMit, 1∶200, Chemicon) were utilized.

Brain sections were incubated in mixed solution of primary antibodies overnight at 4°C as free floating sections, followed by mixed secondary antibodies of Alexa Fluor 488-conjugated anti-mouse IgG (1∶400, Molecular Probe) and Alexa Fluor 594-conjugated anti-rabbit IgG for 1 hr at RT. Negative control sections from each animal were prepared for Immunohistochemical staining in an identical manner except the primary antibodies were omitted. Stained sections were then examined under an Olympus laser confocal fluorescence microscope.

### Stereological cell counts

Experimental animals used in the study are group 1 (control, vehicle/PBS n = 3), group 2 (F3, n = 3) and group 3 (F3.VEGF, n = 3). Total number of human nuclear matrix antigen (HuNuA)-positive F3 and F3.VEGF NSCs in the brain sections from ICH animals was determined by stereological estimation. The sections used for counting covered the entire striatum with hemorrhage lesion and overlying cortex. This generally yielded six or seven sections in a series. Sampling was done using the Computer-assisted stereological toolbox system, version 2.1.4 (Olympus), using an Olympus BX51 microscope, a motorized microscope stage (Prior Scientific, Rockland, NY) run by an IBM compatible computer, and a microcator (Heidenhain ND 281B, Schaumberg, IL) connected to the stage and feeding the computer with the distance information in the z-axis. The counting areas were delineated at a 1.25× objective and generated counting areas of 150×150 µm. A counting frame (1612 µm^2^) was placed randomly on the first counting area and systemically moved through all counting areas until the entire delineated area was sampled. Actual counting was performed using a 100× oil objective. Guard volumes (4 µm from the top and 4–6 µm from the bottom of the section) were excluded from both surfaces to avoid the problem of lost caps, and only the profiles that came into focus within the counting volume (with a depth of 10 µm) were counted. The estimate of the total number of HuNuA-positive F3 and F3.VEGF was calculated according to the optical fractionator's formula [33].

### ELISA assays for human VEGF

F3 and F3.VEGF NSCs were each incubated in medium containing 3% fetal bovine serum for 24 h, and the supernatants were collected, while plastic adherent cells were harvested after a brief incubation in PBS containing 0.25% trypsin and 1 mM EDTA. The cells were lysed in RIPA buffer (150 mM NaCl, 1% Nonidet P-40, 0.5% deoxycholic acid, 0.1% SDS, 50 mM Tris, pH 8.0) containing protease inhibitors, centrifuged at 12,000 rpm for 10 min, and the supernatant was collected. Levels of VEGF in culture supernatants and cell lysates were determined by an ELISA kits specific for human VEGF (R&D System, Minneapolis, MN). Protein concentration was estimated using the method of Bradford [34] with bovine serum albumin as a standard.

ICH mice were sacrificed and decapitated (three groups of seven each, total n = 21) and brains were removed 2 and 8 weeks post-transplantation. Brains were cut in 2 mm coronal sections, frozen and stored at −80°C until the assay. Two-millimeter diameter punches centered on each side of the substantia nigra (SN) were taken according to the atlas of Paxinos and Franklin [35]. Seven tissue samples were collected in each of the three groups from both the ipsillateral and contralateral SN. The collected samples were homogenized with lysis buffer (137 mM NaCl, 20 mM Tris, pH 8.0, 1% NP40, 10% glycerol, 1 mM PMSF, 10 mg/ml aprotinin, 1 mg/ml leupeptin and 0.5 mM sodium vanadate), centrifugation at 12,000 rpm for 10 min and the supernatants were used for ELISA assay.

### Western blot analysis

ICH mice of 2 and 8 weeks post-transplantation were sacrificed and decapitated (three groups of seven each, total n = 21), brains removed, left brain hemisphere containing hemorrhage lesion/cell transplantation sites were dissected out and homogenized in 10 volumes of cold homogenization buffer (50 mM Tris, 120 mM NaCl, pH 7.4) containing protease inhibitors and stored at −80°C. Protein extracts from brain tissue (20 µg) were separated by sodium dodecyl sulfate-polyacrylamide gel electrophoresis. Protein separation was performed using a 10% polyacrylamide with 0.05% bis-acrylamide. Proteins were then transferred to nitrocellulose, and the blots were probed with antibodies specific for VEGF1(1∶1000, Santa Cruz Science, Santa Cruz, CA), VEGF2 (1∶1000, Santa Cruz), caspase 3 (1000, Santa Cruz), Bax (1∶1000, Santa Cruz), Bcl-2 (1∶1000, Santa Cruz), Bcl-xL (1∶1000, Santa Cruz), Akt1 (1∶1000, Chemicon), PI3 kinase p85 and p110 (1∶1000, Oncogene). PI3-kinases catalyze the synthesis of PI-4,5-biphosphate, which regulates various processes including cell proliferation, survival, membrane trafficking, and cytoskeletal organization. PI3-kinase is a heterodymeric complex of an 85K regulatory subunit and an 110K catalytic subunit. Secondary antibodies (Amersham Chicago, IL) and a chemiluminescence kit (Amersham) were used for immunodetection. Western blot analysis was performed on samples from three separated experiments.

### Microvessel count

To investigate the number of microvessels in the ICH brain transplanted with F3 or F3.VEGF human NSCs, coronal brain sections 2 mm anterior and 2 mm posterior from the needle track, were chosen and processed for von Willebrand factor immunostaining. Brain sections were incubated in an antibody specific for von Willebrandt factor (1∶2000, rabiit polyclonal, Oncogene) overnight at RT, followed by Alexa Fluor 594-conjugated anti-rabbit IgG for 1 hr at RT. Number of microvessels in brain sections was determined in a low-power magnification of ×100. The number of microvessels was calculated as the mean of the microvessels counts obtained from the three microscopic images.

### TUNEL staining

Three groups of four mice (total n = 12) were used in the study. DNA fragmentation was detected by using TUNEL POD (Terminal deoxynucleotidyl transferase-mediated d-UTP Nick End Labeling Peroxidase) combined with nonisotopic digoxigenin-11dUTP and terminal transferase according to manufacturer's instructions (Roche Molecular, Indianapolis, IN). Briefly, brain sections were incubated with permeabilization solution (0.1% TritonX-100 in 0.1% sodium citrate) on ice for 2 min, and endogenous peroxidase activity was quenched with 0.3% hydrogen peroxide in methanol for 30 min at RT. After PBS wash, brain sections were incubated with TUNEL reaction mixture for 30 min at 37°C, followed by TUNEL POD for 30 min at RT and the reaction was visualized with DAB reaction.

### Statistical analysis

Data are presented as means±SEM. The statistical significance between group comparisons for behavioral data was determined by one-way ANOVA and two-way ANOVA. P values <0.001 were considered to be statistically significant.

## Results

### Stable human neural stem line overexpressing VEGF

**Figure 2:**
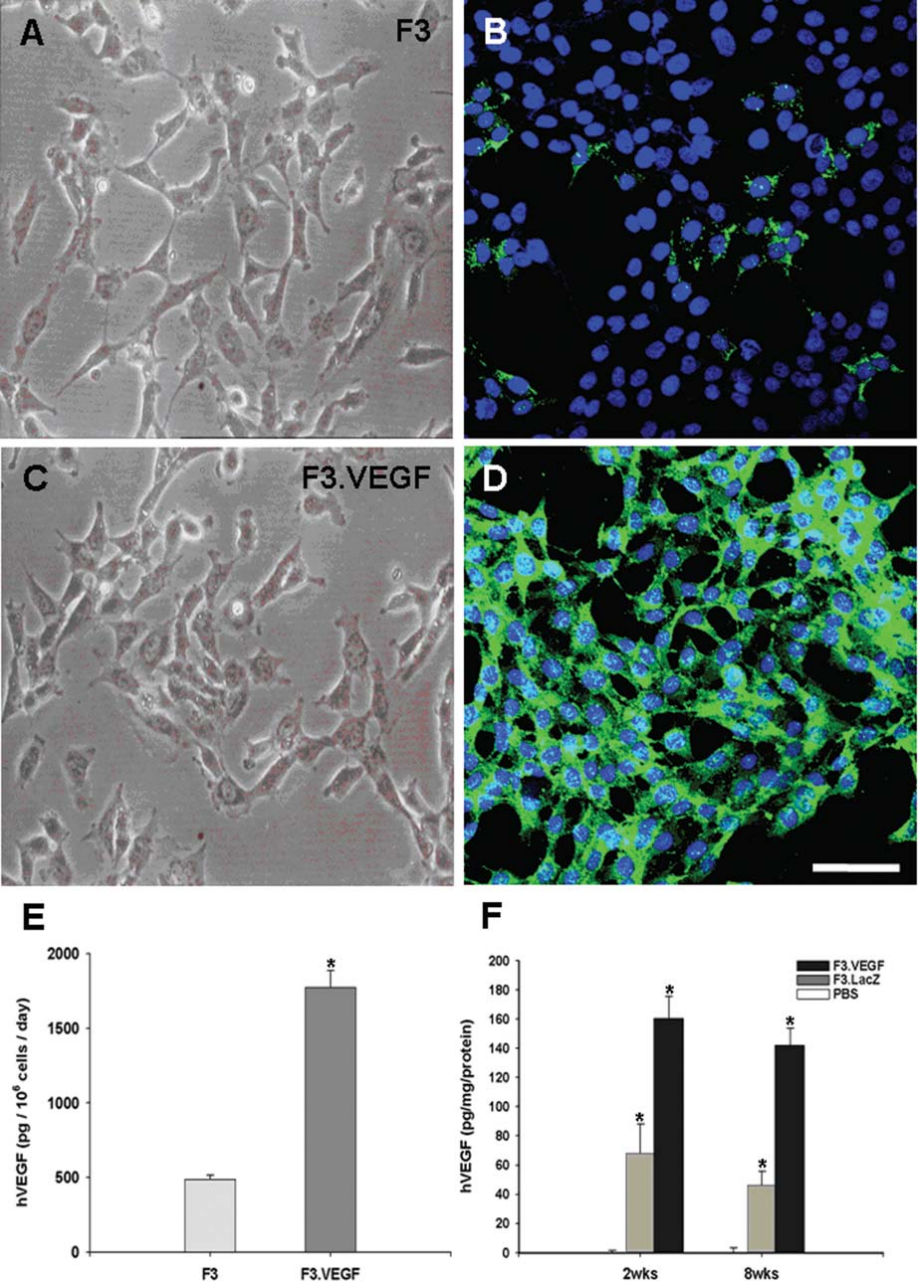
Immunofluorescence microscopy demonstrating production of VEGF in parental F3 human neural stem cell line (A, C) and F3.VEGF, the human neural stem cell line overexpressing VEGF (B, D). Results from the ELISA assay for human VEGF indicate that the levels of VEGF in spent media from F3.VEGF cell line are 5 times over those of F3 cell line (E). Levels of VEGF production in brain sections isolated from intracerebral hemorrhage (ICH) mice grafted with F3 or F3.VEGF human neural stem cells (F). VEGF levels are significantly higher in the F3.VEGF-transplanted mice 2 weeks and 8 weeks post-transplantation as compared with ICH mice grafted with F3 cell line. *: P<0.001, **: P<0.05

HB1.F3 human NSC line was infected with a retroviral vector encoding human VEGF gene twice, clones resistant to puromycin were selected, and then cloned again by limited dilution. The morphology of selected cell line of F3.VEGF does not differ from the parental F3 NSC line with bipolar- or multipolar-morphology (Figs. 2A, C). Immunfluorescence study demonstrated that a small number of parental F3 cells (Fig. 2B) and all the F3.VEGF cells are strongly immunoreactive to VEGF antibody (Fig. 2D).

Results of RT-PCR analysis of mRNAs isolated from F3 and F3.VEGF human NSCs are shown in Figure 1B. Transcripts for nestin (an NSC specific marker), neurofilament triplet proteins (NF-L, NF-M and NF-H, cell type-specific markers for neurons), glial fibrillary acidic protein (GFAP, a specific marker for astrocytes), and VEGF are all expressed by both F3 and F3.VEGF cell lines (Fig. 1B). However, transcript for MBP, structural protein and a specific cell type specific marker for oligodendrocytes, was not demonstrated.

Levels of VEGF in the supernatants of cultured F3 and F3.VEGF cell lines are shown in Fig. 2E. ELISA analyses indicated that human VEGF released by F3.VEGF NSCs in culture media is 3.7-fold over the control F3 parental NSCs at 1832.9±107.3 pg/10^6^ cells/day (mean±SEM) vs 490.8±36.3 pg/10^6^ cells/day (p<0.001). VEGF levels in brain sections prepared from ICH animals transplanted with F3.VEGF, F3 and PBS at 2- and 8-weeks post-transplantation were also determined by VEGF ELISA assay (Fig. 2F). Details of the results are described in a section below (In Vivo Levels of VEGF Production by NSC grafts).

### VEGF expression in transplanted NSCs

**Figure 3:**
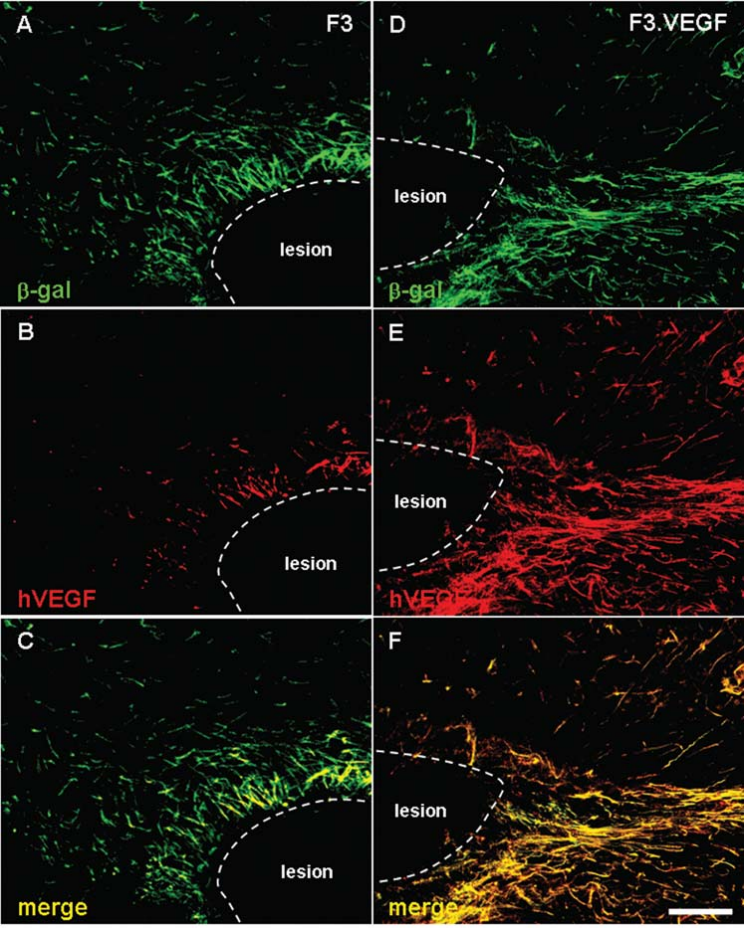
Double immunofluorescence microscopy demonstrating β-gal+/VEGF+ F3 cells (A–C) and β-gal+/VEGF+ F3.VEGF cells (D–F) in the hemorrhage core borders 8 weeks post-transplantation. A markedly higher number of β-gal+/VEGF+ cells were found in the brain region grafted with F3.VEGF cells. Bar indicates 50 µm.

At 7 days after transplantation, F3.VEGF NSCs doubly positive for β-gal and VEGF were found in the hemorrhage core indicating that that the F3.VEGF NSCs were able to migrate from the original injection site to the hemorrhage core and border area, survive and functionally express VEGF at the brain injury sites (Figs. 3A–F). In addition, these F3.VEGF NSCs could migrate long distance and found in further areas in corpus callosum and hippocampal formation (data not shown).

### Functional recovery in ICH animals by NSC transplantation

**Figure 4:**
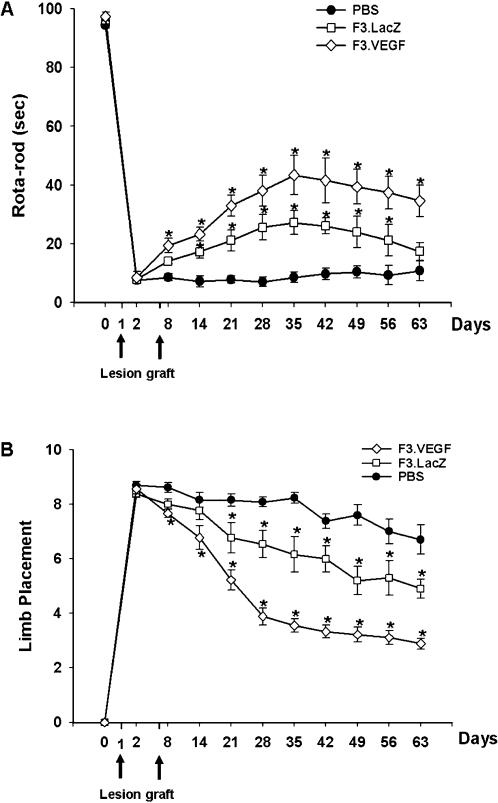
Behavioral improvement demonstrated in mice with intracerebral hemorrhage (ICH) transplanted with F3 or F3.VEGF human neural stem cell lines. A: Rotarod test. F3.VEGF-transplanted group showed better performance than PBS controls or F3-transplanted group from 8 days on, and these benefits continued up to 8 weeks post-transplantation (P<0.001). B: In the modified limb placement test, F3.VEGF-transplanted group showed better performance than PBS or F3-transplanted group (P<0.001).

Motor performance of ICH animals receiving PBS, F3 or F3.VEGF NSCs was determined by the rotarod test and neurology score (Figs. 4A, B). Animals receiving F3.VEGF NSCs were able to remain on the rotarod column significantly longer time than those receiving F3 NSCs (by two-way ANOVA, F = 50.32, P<0.001), while no behavioral recovery was noted in control animals receiving PBS. ICH mice grafted with F3.VEGF NSCs showed a significant improvement in motor performance from 8 days post-transplantation onward and the improvement lasted for up to 8-weeks post-transplantation as compared with controls groups. Significant differences in behavioral performance in F3.VEGF vs F3 groups detected during the period of 3–9 weeks post-transplantation (p = 0.05).

### Survival of transplanted F3 and F3.VEGF NSCs in ICH brain

**Figure 5:**
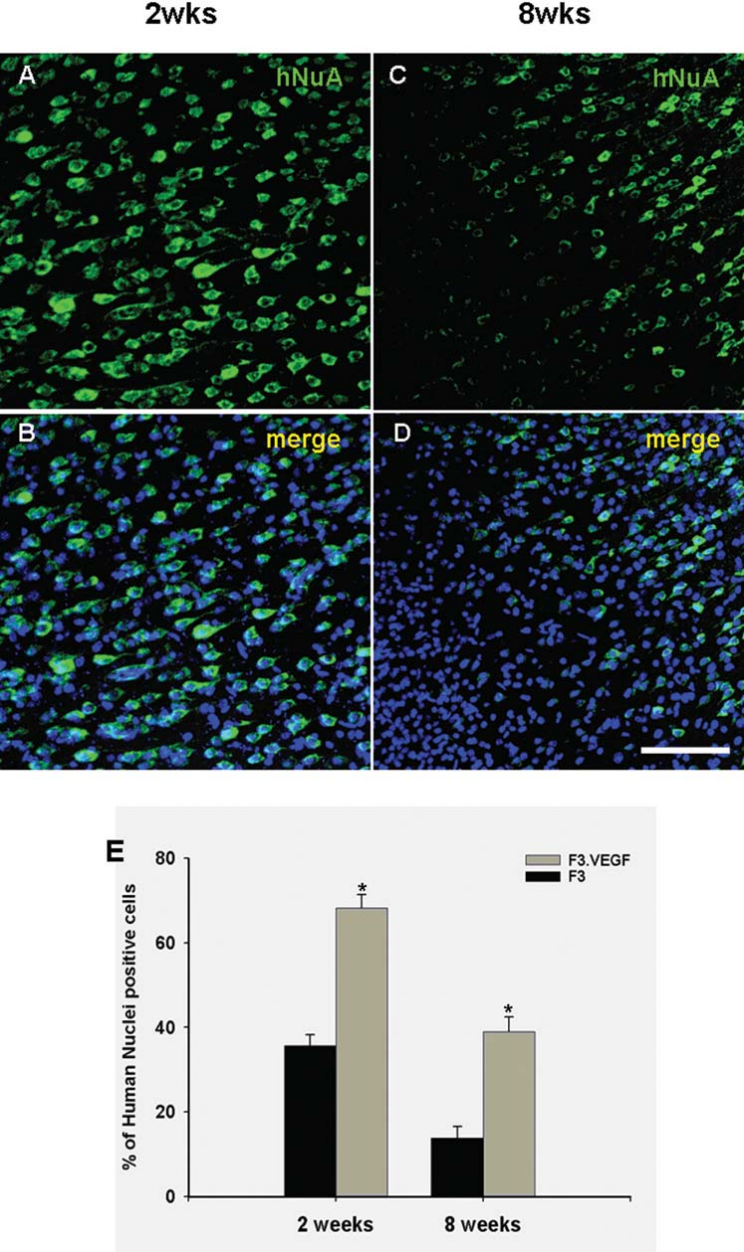
Survival of grafted F3.VEGF human NSCs was demonstrated by immunofluorescence microscopy of human nuclear matrix antigen (HuNuA)/DAPI (A/C-2 weeks, B/D-8 weeks post-transplantation). Number of surviving human NSCs expressing HuNuA (means±SE) in ICH mouse brain at 2 and 8 weeks post-transplantation (E). * P<0.001

At 7 days after induction of experimental ICH, 2×10^5^/2 µl of F3 or F3.VEGF NSCs (both labeled by adeno-LacZ) were transplanted into ICH mouse cerebral cortex overlying hemorrhage lesion site, 2 mm cranial to the hemorrhagic lesion. Total number of LacZ-positive F3 and F3.VEGF NSCs in the brain sections from ICH animals were determined by stereological estimation at 2- and 8-weeks post-transplantation. The results indicate that cell survival at 2-weeks post-transplantations is 136,350±2645 cells (68.2±3.2% of the initial population of 200,000 cells) and at 8 weeks post-transplantation the number is 78,078±2734 cells (39.0±3.3% of the initial population of 200,000 cells) (Figs. 5A–E) These results indicate that the survival rate of grafted F3.VEGF NSCs in the brain of ICH animals is 68% at 2 weeks post-transplantation and 39% at 8 weeks post-transplantation. In ICH animals transplanted with F3 NSCs, the number of surviving cells is 71,297±2171 cells (35.6±13.8% of the initial population of 200,000 cells) at 2-weeks post-transplantation and 27,673±2175 cells (13.8±2.7% of the initial population of 200,000 cells) at 8-weeks (p<0.001). These results indicate that VEGF over-expression in NSCs resulted in a 2-fold increase in cell survival of transplanted NSCs at 2 weeks post-transplantation and a 3-fold increase at 8 weeks post-transplantation. There was no sign of tissue distortion or tumor formation in brain of ICH animals grafted with F3 or F3.VEGF NSCs (data not shown).

### Transplanted NSCs differentiate into neurons and astrocytes

**Figure 6:**
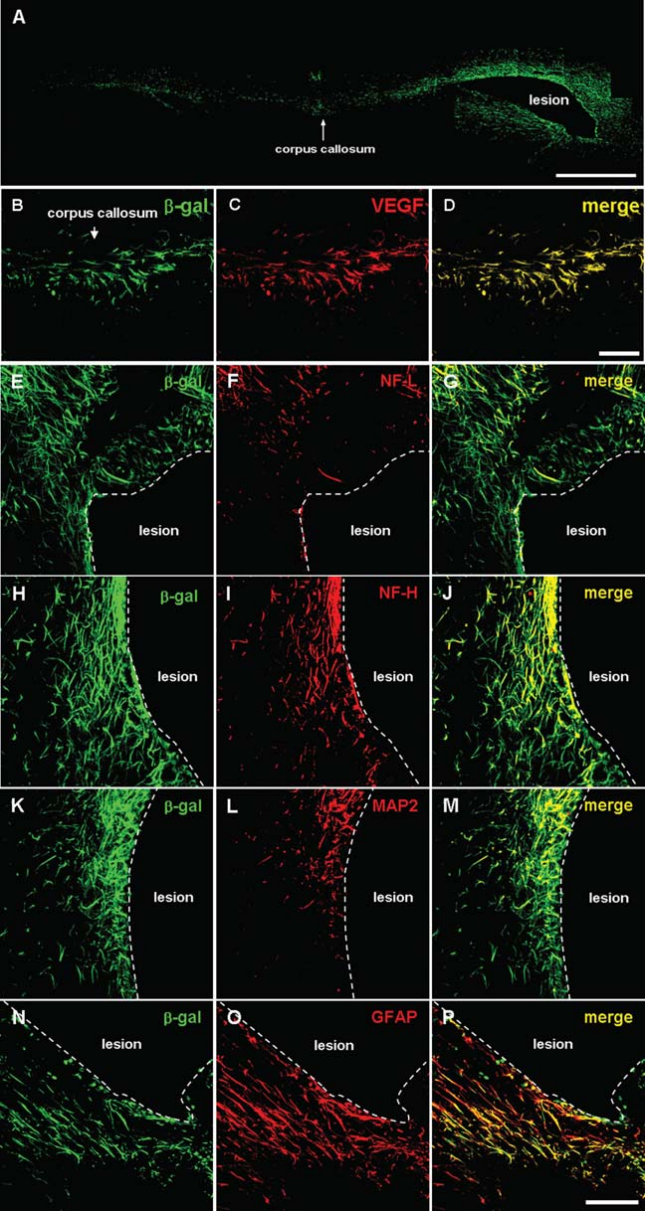
Good survival of F3.VEGF human neural stem cells pre-labeled with adeno-LacZ (β-gal) was found in hemorrhage core or lesion border of experimental ICH mouse brain 8 weeks post-transplantation. A large number of LacZ+ F3.VEGF human neural stem cells were found to migrate to contralateral side of hemisphere via corpus callosum (A). Higher magnification of migrating F3.VEGF cells is also shown (B–D). LacZ+ F3.VEGF cells differentiate into neurons as shown by β-gal+/NF-L+ (E–G), β-gal+NF-H+ (H–J) and β-gal/MAP2 (K–M) and also into astrocytes as demonstrated by β-gal+/GFAP+ staining (N–P). Bar indicates 50 µm.

Following transplantation into ICH mouse cerebral cortex overlying hemorrhage lesion site, β-gal+/LacZ+ human NSCs migrated selectively to the hemorrhagic core and also located on the border of the lesion and further away from the injection sites. We also found β-gal-positive F3.VEGF NSCs to the other brain sites including corpus callosum and hippocampus (Figs. 6A–D). A large number of transplanted β-gal+ F3.VEGF cells (35–45%) differentiated into NF-L+/NF-H+ (Figs. 6E–J) or MAP2+ (Figs. 6K–M) neurons in the peri-hematomal sites. Similarly a large proportion of the transplanted β-gal+ F3.VEGF cells (55–65%) were GFAP+ astrocytes (Figs. 6N–P). Many of the β-gal+/GFAP+ double-positive cells were found along the border of hemorrhagic core. These results indicate that a majority of grafted F3.VGEF cells differentiate into either neurons or astrocytes in response to signals from the local microenvironment provided by the hemorrhagic lesion.

### F3.VEGF stimulates proliferation of host microvessels

**Figure 7:**
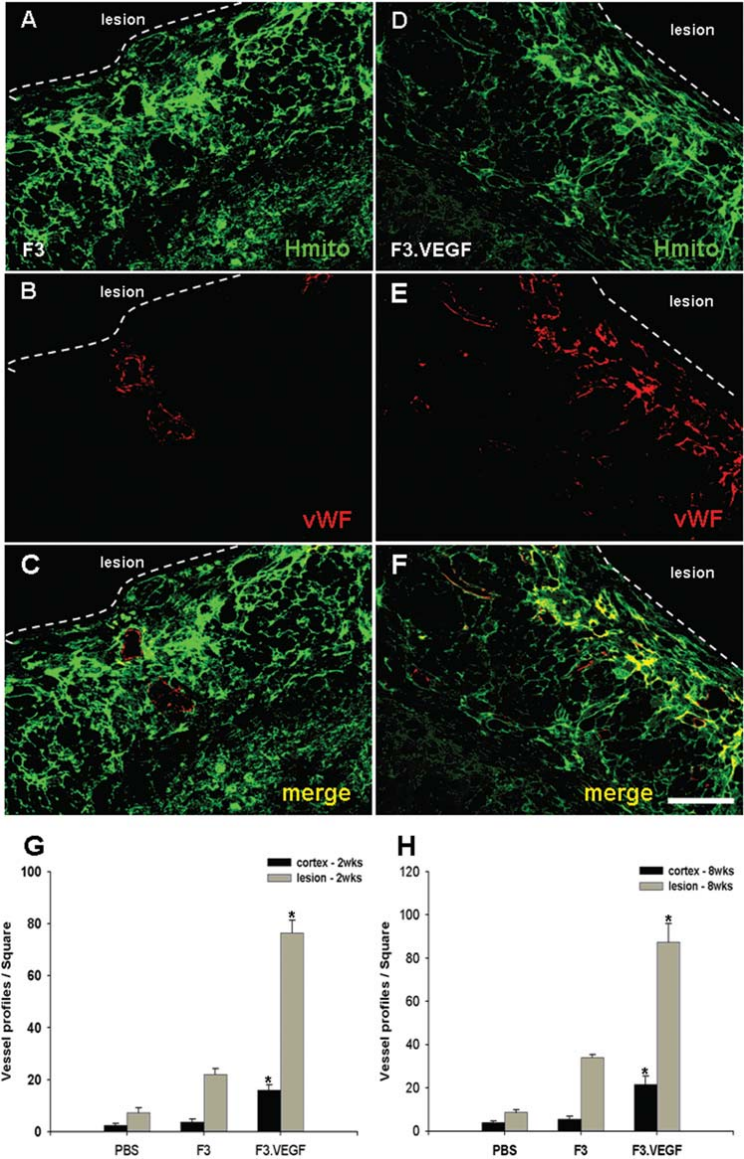
A promoted angiogenesis in hemorrhage lesion by transplanted F3 (A–C) and F3.VEGF (D–F) human NSCs was demonstrated by double immunofluorescence staining of human motochondria (HuMit, green) and von Willebrandt factor (vWF, red). Number of microvessels immunoreaction-positive for von Willebrandt factor is significantly increased in F3.VEGF group as compared with control F3 group. Bar indicates 50 µm. * P<0.001

To determine if transplanted F3.VEGF NSCs promote proliferation of host endothelial cells/microvessels in the lesion sites, number of endothelial cells 8 weeks post-transplantation was determined using von Willebrandt factor (vWF) immunostaining. Results indicated that the number of vWF-positive microvessels in the F3.VEGF group was significantly higher as compared with those in the F3 and PBS groups (Figs. 7A–F). Count of vWF-positive microvessels in ICH brain sections indicates that there is 3–4 fold increase in vWF-positive microvessels in the brain receiving F3.VEGF cells as compared to the control F3 cells at 2 and 8 weeks post-transplantation (Figs. 7G, H).

### TUNEL staining

**Figure 8:**
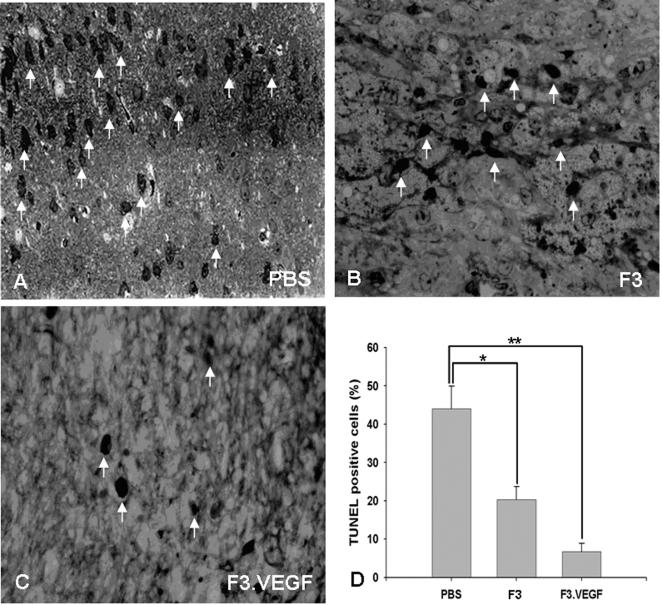
At 8 weeks post-transplantation in the hemorrhage core border areas, the number of TUNEL (in situ end-labeling of nuclear DNA fragmentation)-positive cells was highest in PBS-ICH control animals (A), while the number of TUNEL-positive cells is much lower in the ICH-F3 (B) or ICH-F3.VEGF groups (C). Bar indicates 100 µm.

A large number of TUNEL (in situ end-labeling of nuclear DNA fragmentation)-positive cells were found in the brain sections in the PBS-ICH control animals. At 8 weeks post- transplantation in the hemorrhage core border areas, the number of TUNEL-positive cells was much lower in the ICH animals receiving transplantation of F3.VEGF cells than ICH animals receiving PBS or F3 cells (Fig. 8A–D). The number of TUNEL-positive cells in ICH/F3.VEGF brain was similar in the lateral or medial borders. This pattern was similar in ICH mice grafted with F3 cells. Interestingly, the number of TUNEL-positive cells bearing nuclear DNA fragments was significantly reduced in the medial border of the hemorrhage core, which was in the vicinity of the F3.VEGF grafts, but not in the lateral border, away from the NSC grafts.

### In vivo levels of VEGF production by NSC grafts

VEGF levels in brain sections prepared from ICH animals transplanted with F3.VEGF, F3 and PBS at 2- and 8-weeks post-transplantation were determined by VEGF ELISA assay (Fig. 4A). VEGF levels in F3.VEGF-transplanted brain loci were 156.9±18.6 pg/mg protein (2 weeks) and 138.5±15.2 pg/mg proteins (8 weeks), while in the brain sections of F3 grafted loci was 67.9±20.0 pg/mg (2 weeks) and 46.2±9.5 pg/mg (8 weeks). In PBS-injected ICH animals, VEGF contents were below the detectable level of the VEGF ELISA assay. These results indicate that F3.VEGF NSCs at the ICH lesion sites are capable of producing markedly higher amount of VEGF than the parental F3 cells in the lesion sites and thus capable of providing neuroprotective and neoangiogenetic action in the ICH injury sites resulting in improved behavioral outcome. VEGF levels in ICH animals grafted with F3.VEGF, F3 or PBS were also determined by Western blot analyses. ICH animals with F3.VEGF cell transplantation showed higher levels of VEGF than animals receiving F3 cells (Data not shown). These results agree closely with results obtained with immunocytochemical VEGF staining (Fig. 3).

### VEGF blocks apoptotic cell death

**Figure 9:**
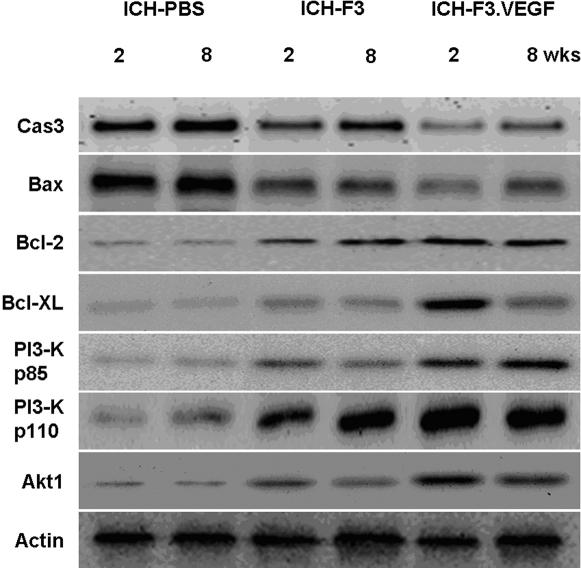
Western blot analysis of apoptosis-related proteins, including proapoptotic proteins, (caspase 3 and Bax), antiapoptotic proteins (Bcl-2 and Bcl-X_L_), and cell survival signal molecules (PI3-kinase p85, PI3 kinase p110 and Akt1) in ICH brains receiving transplantationof F3 and F3.VEGF human neural stem cells. A significantly decreased expression of caspase 3 and bax is found in ICH-F3.VEGF group at 2 and 8 weeks post-transplantation. In ICH animals receiving F3.VEGF grafts, the expression of antiapototic proteins and signal molecules Bcl-2, Bcl-X_L_, PI3-K p85, PI3-K p110 and Akt1 is markedly increased.

To understand the mechanisms underlying neuroprotecitive action of VEGF in the ICH brain, we examined expression of several apoptosis-related proteins including pro-apoptotic proteins caspase 3 and Bax, anti-apoptotic proteins Bcl-2 and Bcl-X_L_, and survival signal molecules p85, p110 and Akt1 using Western blot analyses. The results showed that the expression of proapoptotic protein caspase 3 and Bax increased in the ICH-PBS group, while in ICH brain receiving-F3 and ICH-F3.VEGF group marked reduction in their expression was found (Fig. 9). In converse to the levels of proapoptotic proteins, levels of antiapoptotic proteins Bcl-2, Bcl-X_L_ and survival signal molecules p85, p110 and Akt1 in ICH brain receiving F3 or F3.VEGF grafts were significantly higher as compared with ICH-PBS control group (Fig. 9). These results suggest that F3.VEGF provides neuroprotective effect via down-regulation of expression in pro-apoptotic proteins while production of anti-apoptotic proteins and cell survival promoting molecules is up-regulated.

## Discussion

In the present study, an animal model of intracerebral hemorrhage (ICH) was used to provide proof-of-principle that human NSCs over-expressing growth factor VEGF can be transplanted in the brain of animal models of neurological diseases, and produce beneficial effects of functional recovery, increased angiogenesis in the host brain and increased survival of grafted NSCs. We also tested the hypothesis that the human NSCs could be used as a novel carrier for the delivery of the VEGF gene, providing neuroprotection in animals suffering from ICH injury. Our results demonstrated that the F3 human NSCs transduced with VEGF gene could survive well in the ICH animal brain and release VEGF protein in the hemorrhagic brain injury sites following transplantation.

In the present study, from as early as one day post-transplantation to 8 weeks post-transplantation, F3.VEGF cells provided functional recovery as determined by rotarod test and limb placement test and also induced an increased survival of transplanted NSCs, inhibition of apoptosis, and renewed angiogenesis in the host brain. The present study demonstrates that VEGF secreted from transplanted human NSCs promoted cell survival pathways and blocked cell death in the ICH brain.

F3 NSCs overexpressing VEGF were able to survive much better than parental F3 NSCs, so that there were a 2-fold increase in cell survival of transplanted F3.VEGF cells at 2 weeks post-transplantation and a 3-fold increase at 8 weeks, and a majority of grafted F3.VGEF cells differentiated into either neurons or astrocytes in response to signals from the local microenvironment. In addition, the number of TUNEL-positive cells, neruons undergoing apoptotic cell death, was much lower in the ICH animals receiving F3.VEGF cells than in animals receiving PBS or parental F3 cells. The number of von Willebrandt factor-positive microvessels in ICH brain receiving F3.VEGF cells is 3–4 fold higher as compared to the control F3-ICH brain. These results indicate that F3.VEGF NSCs at the ICH lesion sites are capable of producing markedly higher amount of VEGF than the parental F3 cells and thus providing neuroprotective and neoangiogenetic action in the ICH injury sites resulting in improved behavioral outcome.

The present results showed a marked reduction in expression of proapoptotic proteins caspase 3 and Bax in ICH brain receiving F3 or F3.VEGF cells, while their expression was found to increase in control vehicle/PBS group. In converse to the levels of proapoptotic proteins, levels of antiapoptotic proteins (Bcl-2 and Bcl-xL) and survival signal molecules (p85, p110 and Akt1) in ICH brain receiving F3 or F3.VEGF grafts were significantly higher as compared with control ICH-PBS group. These results suggest that F3.VEGF cells provide neuroprotective effect via down-regulation of expression in pro-apoptotic proteins while production of anti-apoptotic proteins and cell survival promoting molecules are up-regulated. It is known that VEGF activates PI3-kinase-Akt pathway via VEGF receptors. Akt, a serine−threonine protein kinase, exerts antiapoptotic effects by preventing the release of cytochrome c from mitochondria, by inactivating fork-head transcription factors by which transcription of proapoptotic genes is inhibited, by inducing transcription of the survival genes regulated by NF-κB and CREB, and by phosphorylating and inactivating the proapoptotic factors BAD and pro-caspase-9 [36]–[38]. As demonstrated in the present study, VEGF is also beneficial to the ICH-injured brain indirectly by promoting angiogeneis. New vessel formation mediated by VEGF will improve blood supply to the injured region, thereby efficiently providing oxygen and nutrients to the degenerating and regenerating neurons in the injured brain.

Previous studies have demonstrated that the VEGF treatment in vivo and in vitro could provide protection against several forms of neuronal injury independently of its angiogenic action. For example, VEGF enhances the survival of mouse hippocampal neurons subjected to in vitro ischemia [39], and glutamate or N-methyl-D-aspartate (NMDA) excitotoxicity [40], [41]. In a middle cerebral artery (MCA) occlusion ischemia model, VEGF was found to induce proliferation and survival of endothelial cells under the ischemic injury [42]. Several previous studies have reported that the infusion of VEGF into ischemia lesioned brain produced somewhat inconsistent results [24]–[28], [43]. In earlier studies, VEGF induces blood-brain barrier (BBB) break down and vascular leakage resulting in brain edema [25], [43]. Similarly, application of adenovirus-VEGF in the ischemic brain caused an inflammatory response and BBB disruption [44]. In contrast, application of exogenous VEGF has shown direct neuroprotection and decreased lesion volume independent of angiogenetic activity [24], [26]–[28]. These differences in outcome could be attributed to different route of VEGF administration, concentrations and amount of VEGF used, and expression of VEGF receptors [45]. In comparison with intracerebral or intracerebroventricular application of exogenous VEGF as previous studies have reported, intracerebral transplantation of human NSCs producing VEGF, as shown in this study, is reproducible, provides a unique way to supply sufficient amount of VEGF continuously, produce neuroprotection, and stimulate neurogenesis and angiogenesis in the ICH brain.

The neuroprotective effect of VEGF in ICH mice is mediated by VEGF's ability to promote neuronal survival by active inhibition of apoptosis, which is accomplished by inhibiting the expression of pro-apoptotic factors as well as promoting the expression of antiapoptotic factors [27], [46]. PI3 kinase pathway, activated by many survival factors, leads to the activation of Akt, an important player in survival signaling pathways. Activated Akt inhibits the pro-apoptotic factors Bad, caspase-9, GSK-3 and FKHR by phosphorylation. Growth factors such as VEGF induce anti-apoptotic BCl-2 family members that protect the integrity of mitochondria, preventing cytochrome C release and the subsequent activation of caspase-9 and -3 [47]–[49]. A recent study has shown that VEGF can also act on the neuronal microtubular content, which is involved with growth, stability and maturation of neurons [50]. Taken together, the present study demonstrates that the transplanted F3.VEGF human NSCs provide neuroprotective effect in ICH mouse brain via downregulation of pro-apoptotic protein expression and upregulation of anti-apoptotic proteins and cell survival promoting molecules.

In conclusion, our study suggests that in the ICH mouse brain, transplantation of VEGF-producing human NSCs (a) provides neuroprotection against hemorrhage injury, (b) improves functional recovery in ICH animals, and (c) stimulates angiogenesis in ICH lesion and border areas. These results suggest a possible application of the human neural stem cell line which is genetically modified to overexpress VEGF as a therapeutic agent for ICH-stroke.
